# Supplementary material for: Synthesis of Multicolor Carbon Dots Based on Solvent Control and Its Application in the Detection of Crystal Violet
Source: Nanomaterials (Basel). 2019 Nov 1;9(11):1556. doi: 10.3390/nano9111556 (PMC6915586; doi:10.3390/nano9111556)
Supplement: Supplementary file 1 [file nanomaterials-09-01556-s001.pdf]

Supplementary Materials: Synthesis of Multicolor Carbon Dots Based on Solvent Control and Its Application in the Detection of Crystal Violet

Dan Zhao*, Xuemei Liu, Zhixia Zhang, Rui Zhang, Liangxiu Liao, Xincai Xiao and Han Cheng

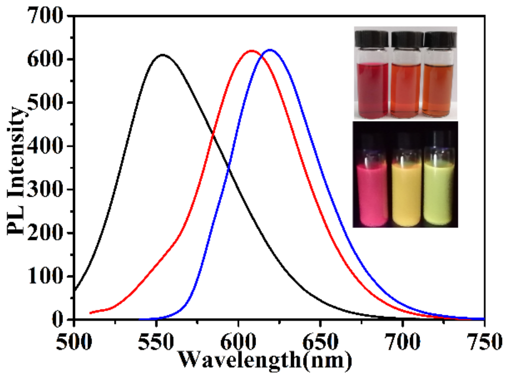

Figure S1. R-CDs Fluorescence spectra of four samples obtained by separation.

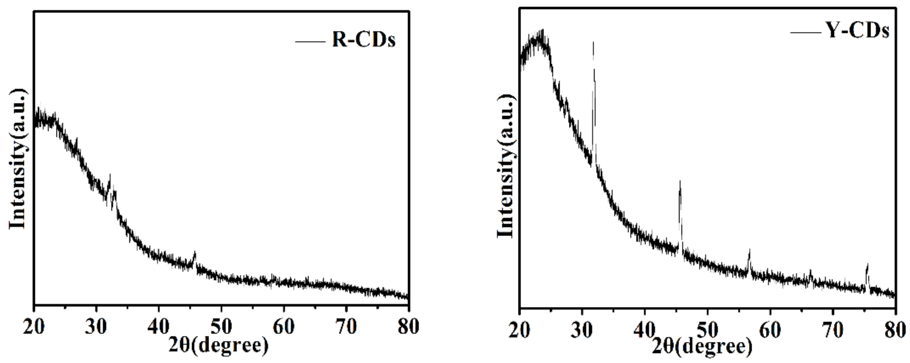

Figure S2. XRD patterns of the R-CDs and Y-CDs.

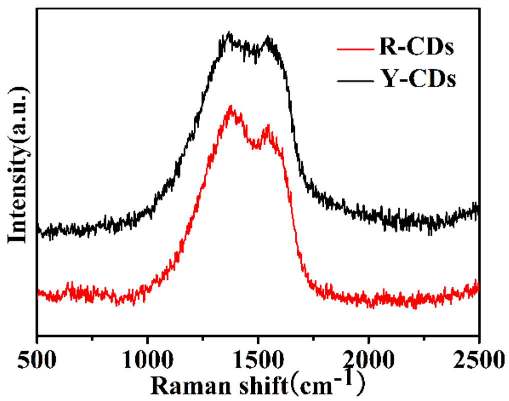

Figure S3. Raman spectra of the R-CDs and Y-CDs.

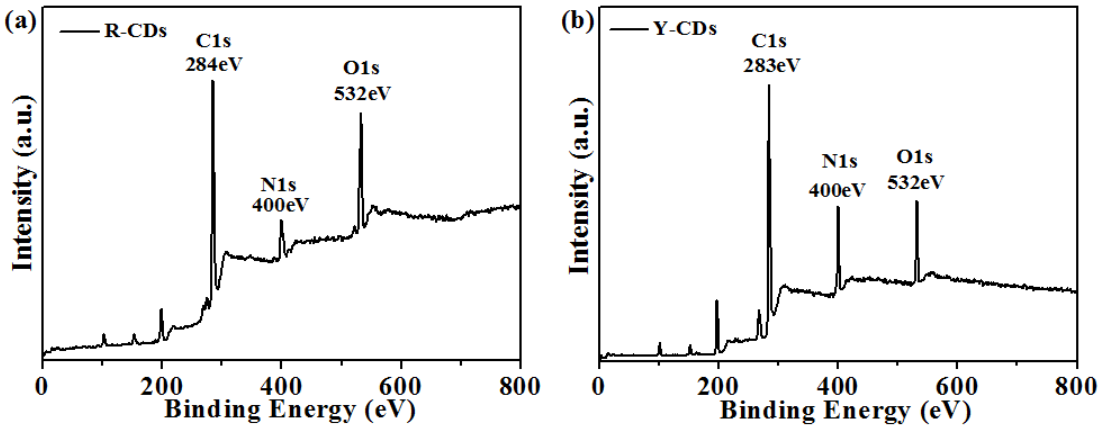

Figure S4. (**a**) R-CDs and (**b**) Y-CDs with XPS full spectrum.

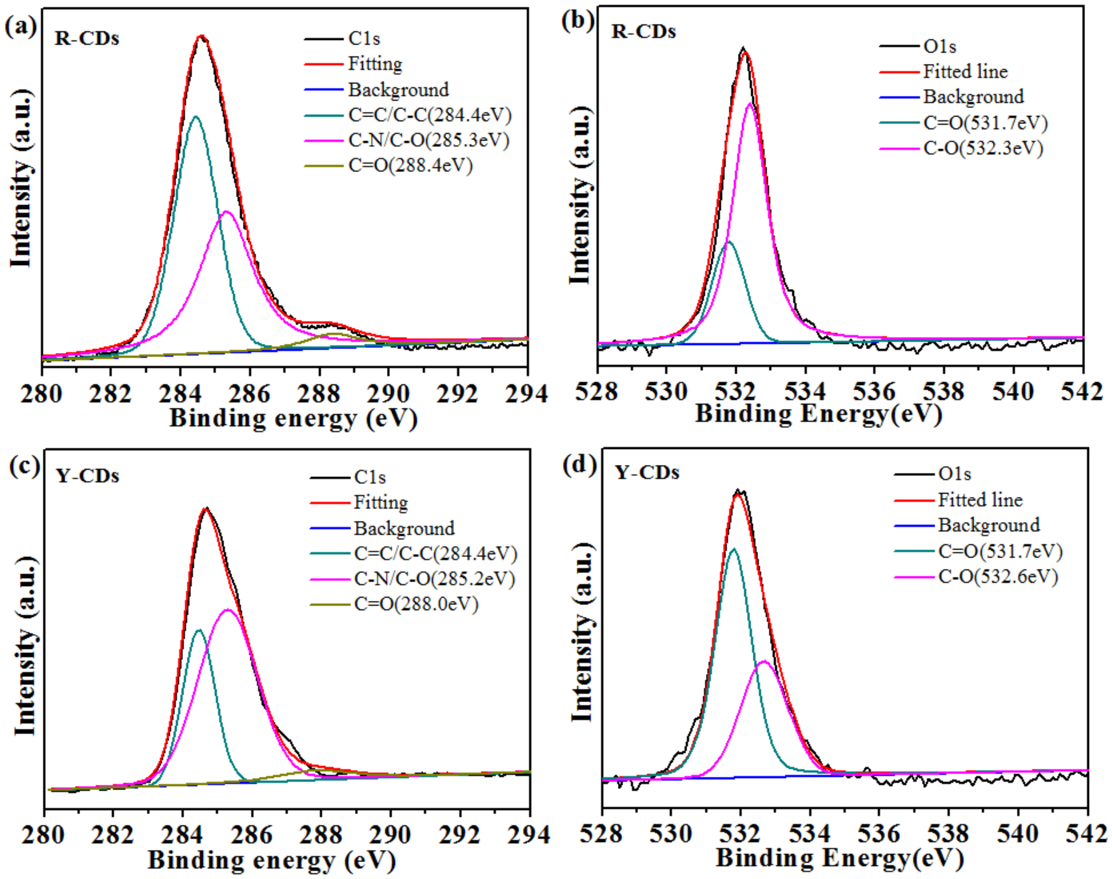

Figure S5. (**a**) C1s and (**b**) O1s spectra of R-CD, (**c**) C1s and (**d**) O1s spectra of Y-CDs.

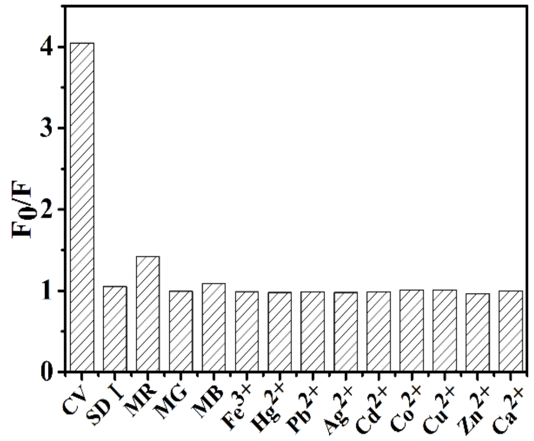

Figure S6. Sensitivity investigation of the Y-CDs for CV detection (samples concentration: 10 μM).

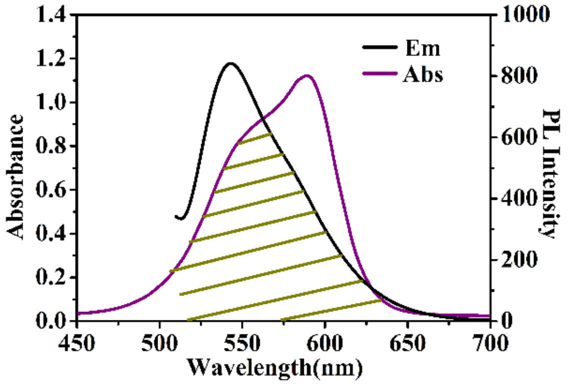

Figure S7. The overlap between absorption spectrum of CV and the emission spectra of Y-CDs.
